# Underdiagnosed CKD in Geriatric Trauma Patients and Potent Prevention of Renal Impairment from Polypharmacy Risks through Individual Pharmacotherapy Management (IPM-III)

**DOI:** 10.3390/jcm12134545

**Published:** 2023-07-07

**Authors:** Ursula Wolf, Hassan Ghadir, Luise Drewas, Rüdiger Neef

**Affiliations:** 1 Pharmacotherapy Management, University Hospital Halle (Saale), Martin Luther University Halle-Wittenberg, 06120 Halle (Saale), Germany; 2 Medical Clinic II, University Hospital Schleswig-Holstein, Lübeck Campus, 23562 Lübeck, Germany; 3 Internal Medicine Clinic II, Martha-Maria Hospital Halle-Dölau, 06120 Halle (Saale), Germany; 4 Department of Orthopedics, Trauma and Reconstructive Surgery, Division of Geriatric Traumatology, University Hospital Halle (Saale), Martin Luther University Halle-Wittenberg, 06120 Halle (Saale), Germany; ruediger.neef@uk-halle.de

**Keywords:** chronic kidney disease (CKD), underdiagnosed, iatrogenic risk, pharmacovigilance, geriatric patients, multimorbidity, polypharmacy, acute kidney injury (AKI), prevention, electronic patient records, medication review, individual pharmacotherapy management, adverse drug reactions (ADRs), drug-drug interactions (DDIs), overdosage, patient safety, drug safety, socioeconomic healthcare system burden

## Abstract

The aging global patient population with multimorbidity and concomitant polypharmacy is at increased risk for acute and chronic kidney disease, particularly with severe additional disease states or invasive surgical procedures. Because from the expertise of more than 58,600 self-reviewed medications, adverse drug reactions, drug interactions, inadequate dosing, and contraindications all proved to cause or exacerbate the worsening of renal function, we analyzed the association of an electronic patient record- and Summaries of Product Characteristics (SmPCs)-based comprehensive individual pharmacotherapy management (IPM) in the setting of 14 daily interdisciplinary patient visits with the outcome: further renal impairment with reduction of eGFR ≥ 20 mL/min (redGFR) in hospitalized trauma patients ≥ 70 years of age. The retrospective clinical study of 404 trauma patients comparing the historical control group (CG) before IPM with the IPM intervention group (IG) revealed a group-match in terms of potential confounders such as age, sex, BMI, arterial hypertension, diabetes mellitus, and injury patterns. Preexisting chronic kidney disease (CKD) > stage 2 diagnosed as eGFR < 60 mL/min/1.73 m^2^ on hospital admission was 42% in the CG versus 50% in the IG, although in each group only less than 50% of this was coded as an ICD diagnosis in the patients’ discharge letters (19% in CG and 21% in IG). IPM revealed an absolute risk reduction in redGFR of 5.5% (11 of 199 CG patients) to 0% in the IPM visit IG, a relative risk reduction of 100%, NNT 18, indicating high efficacy of IPM and benefit in improving outcomes. There even remained an additive superimposed significant association that included patients in the IPM group before/beyond the 14 daily IPM interventions, with a relative redGFR risk reduction of 0.55 (55%) to 2.5% (5 of 204 patients), OR 0.48 [95% CI 0.438–0.538] (*p* < 0.001). Bacteriuria, loop diuretics, allopurinol, eGFR ≥ 60 mL/min/1.73 m^2^, eGFR < 60 mL/min/1.73 m^2^, and CKD 3b were significantly associated with redGFR; of the latter, 10.5% developed redGFR. Further multivariable regression analysis adjusting for these and established risk factors revealed an additive, superimposed IPM effect on redGFR with an OR 0.238 [95% CI 0.06–0.91], relative risk reduction of 76.2%, regression coefficient −1.437 including patients not yet visited in the IPM period. As consequences of the IPM procedure, the IG differed from the CG by a significant reduction of NSAIDs (*p* < 0.001), HCT (*p* = 0.028) and Würzburger pain drip (*p* < 0.001), and significantly increased prescription rate of antibiotics (*p* = 0.004). In conclusion, (1) more than 50% of CKD in geriatric patients was not pre-recognized and underdiagnosed, and (2) the electronic patient records-based IPM interdisciplinary networking strategy was associated with effective prevention of further periinterventional renal impairment and requires obligatory implementation in all elderly patients to urgently improve patient and drug safety.

## 1. Introduction

Health care management in a global demographic context means treating the increasing numbers of geriatric patients worldwide with multimorbidity and polypharmacy. This interconnects with serious risks requiring the earliest possible preventive measures against e.g., renal failure, fall events, and cognitive impairment. The appropriateness of medication therapy for older people and the issues involved in drug and patient safety are a challenge that WHO continues to address [1,2,3], and, e.g., corresponding UN [4,5] and OECD resolutions [6] further weight the recognized patient-related burden and socioeconomic health costs. Within the proclaimed Decade of Healthy Ageing from 2020 to 2030, the number of people aged ≥60 years will increase by 34% from 1 billion in 2019 to 1.4 billion, and by 2050, be doubled to 2.1 billion [7].

Unavoidable polypharmacy in multimorbidity, including age-related renal impairment and more prevalent chronic kidney disease (CKD), accordingly requires pharmacovigilance with a rigorous focus on the exclusion of any iatrogenic, drug-induced decline in renal function. In particular, the preexisting patient condition in combined organ deterioration and polypharmacy poses a high risk of drug-induced complications as caused by adverse drug reactions (ADRs), pharmacodynamic and pharmacokinetic drug-drug interactions (DDIs), overdose, and contraindications in these most vulnerable elderly and very old patients, notably in acute illnesses and in the perioperative course, which per se implies additional drug treatment, namely, narcotics, analgesics, antithrombotics, and antimicrobials. These risks turned out to be of high prevalence as learned from the own conceptualized and conducted, most comprehensive individual pharmacotherapy management (IPM) through >58,600 medication reviews. 

The individual results of the IPM polypharmacy insights are alarming, especially in elderly patients who suffer from medication-induced, iatrogenic cognitive decline, delirium [8], fall events, oropharyngeal dysphagia [9], and organ deteriorations [10] with patient’s acute and long-term burden and elementary financial impact on the socioeconomic health care system worldwide. US Centers for Disease Control and Prevention (CDC) outline CKD relevant facts as being a leading cause of death, more than 1 in 7, 15% (about 37 million) of US adults are estimated to have CKD most undiagnosed, even 40% of people with severe renal impairment are not aware of having CKD. Total Medicare costs for people with CKD were USD 87.2 billion in 2019, corresponding to USD 24,453 per Medicare beneficiary older than 65 years. And the entire Medicare expenditures (including prescription drugs) for end-stage renal disease (ESRD) or kidney failure patients were USD 37.3 billion, USD 86,400 per person, respectively [11]. For Europe according to the CaReMe CKD study including 11 European countries the pooled prevalence of possible CKD is 10% [12]. In Germany, the prevalence of chronic renal dysfunction with an estimated GFR (eGFR) < 60 mL/min/1.73 m^2^ or urinary albumin excretion ≥ 30 mg/l for persons over 70 years of age has been reported to be approximately 28% [13]. The Berlin Initiative Study (BIS)1 equation even verifies a 50% prevalence of GFR < 60 mL/min/1.73 m^2^ in their mean 78.5-year-old study group [14]. 

This is a relevant number, as although demographic aging is perceived to occur gradually, it will accelerate significantly in the near future. In addition, the number of people from the old age of 80 will rise sharply [15,16]. As these elderly patients are particularly vulnerable with regard to iatrogenic renal injury, the long overdue preventative approach has to capture all kinds of eliminable acute and long-term medication risks in this context.

The aim of this single-center study was to analyze whether the implementation of IPM might be associated with the reduction in a further decline in renal function in elderly hospitalized patients on polypharmacy throughout the susceptible perioperative course. 

This study is the third research issue in a comprehensive series of evaluative analyses of the effectiveness of the electronic patient record-based IPM with congruent methodological and patient baseline data, respectively [8,17,18].

## 2. Methods

### 2.1. Study Design and Patients

A retrospective clinical study at the Halle University Hospital (UKH) was conducted on 404 elderly in-hospital patients ≥ 70 yrs of age admitted to the trauma department. We focused on the outcome: additional decline in renal function during hospitalization, defined as a further reduction in eGFR of ≥20 mL/min (redGFR) compared to eGFR at admission and means any reduction in this amount during hospitalization, independent of preceding kidney function. With respect to the geriatric study population, we preferred eGFR as the more accurate outcome to assess. In elderly patients, who typically have lower muscle mass, the definition of acute kidney injury (AKI) by an increase in serum creatinine has been considered less reliable [19], and urine volume is not routinely measured in the trauma department. A patient control group (CG) without IPM from the time period before IPM implementation (2/2009–12/2010) was compared to the intervention group from the IPM period (IG) (5/2012–8/2016) hospitalized and treated in the same ward. In the Department of Trauma Surgery, the total number of geriatric patients aged ≥70 years admitted as inpatients in the years of recruitment was: 335 patients in 2009 and 459 patients in 2010, 433 patients in 2012, 477 in 2013, 471 in 2014, 428 in 2015, and 431 in 2016. Recruitment of patients in the intervention group over the years was intended to assess lasting and stable potential effects. The random patient recruitment, blinded for the outcome redGFR, provided a cross-matched study population from the non-proactive group matches in terms of age, gender, residency, BMI, CKD-relevant diagnoses of diabetes mellitus and arterial hypertension, and injury patterns, thus providing an important prerequisite for group comparison by the exclusion of these most relevant potential confounders.

### 2.2. Clinical Setting and Data Collection

IPM for inpatients ≥ 70 years of age started in February 2011 at the UKH Department of Trauma Surgery with the implementation of interdisciplinary fortnightly ward rounds on the geriatric traumatology ward including individual medication reviews. Uniform personal consistency of patient visits was ensured by the constant presence of the same specialist in charge of internal medicine/pharmacotherapy management and the same senior physician in geriatric traumatology, accompanied by residents and geriatricians, nurses, and medical students. There were no discernible changes in perioperative medical or nursing management over time relevant to the analysis, including the spectrum of trauma and fractures that remained almost constant.

The entirety of the in-hospital digital patient records enabled a comprehensive data collection of interest in the context of this study and further issues addressed [8,17], and was analogously also the basis for the integrated patient scores in the process of the implemented IPM (Table 1).

The eGFR was measured by the UKH laboratory. We assessed the primary renal function of each patient from the actual eGFR as a more accurate parameter compared to ICD-coded renal function from the diagnosis in the patients’ medical discharge letters which often missed the renal impairment. For most patients, there was no information in terms of the duration of kidney disease and there is no regular focus on albuminuria in a trauma surgery department.

Until the onset of this retrospective study, at UKH the eGFR was determined according to the Modification of Diet in Renal Disease Study (MDRD) formula, developed 1999 [20]. Accordingly, patients with CKD 1 and CKD 2 were not differentiated as in total covered by eGFR ≥ 60mL/min. Serum creatinine, age, sex, and race (ASR) were established as standard variables in the equation, and a body surface area of 1.73 m^2^. The MDRD formula is more accurate than the Cockcroft-Gault formula in CKD patients with moderate to severe stages [20]: eGFR [mL/min/1.73 m^2^] = 186 × (creatinine[mg/dL]exp(−1.154)x age[years] exp(−0.203) correction factor * (* Correction factor: for women, 0.742). And as a consequence of the study, now the UKH laboratory has been using the CKD-EPI equation for eGFR since 2016, which affects some of the later IG patients. The differentiated arguments for the use of the various estimated GFRs are of practical and clinical relevance [21,22,23]. We additionally assessed eGFR by applying the BIS-1 formula.

### 2.3. Individual Pharmacotherapy Management (IPM)

IPM was conceptualized and conducted by a single UKH physician, who specialized in internal medicine, had six years of additional expertise in nephrology and kidney transplantation, and was further educated in clinical pharmacology. On this educational background the IPM medication reviews thus always result from a synoptic internistic/clinical pharmacologic view and have started implementation by regularly ongoing 14 daily interdisciplinary and interprofessional patient ward rounds in the division of geriatric traumatology, UKH, in 2011.

The primary intention of the IPM concept is to take the most comprehensive overall view of each patient in his very individual clinical condition for optimized adaption of every single drug from the entire medication list according to the drugs’ summaries of product characteristics (SmPCs), guidelines, DDI checks [24], dosing recommendations in renal impairment [25], and additional PubMed literature research if required. 

**Figure 1:**
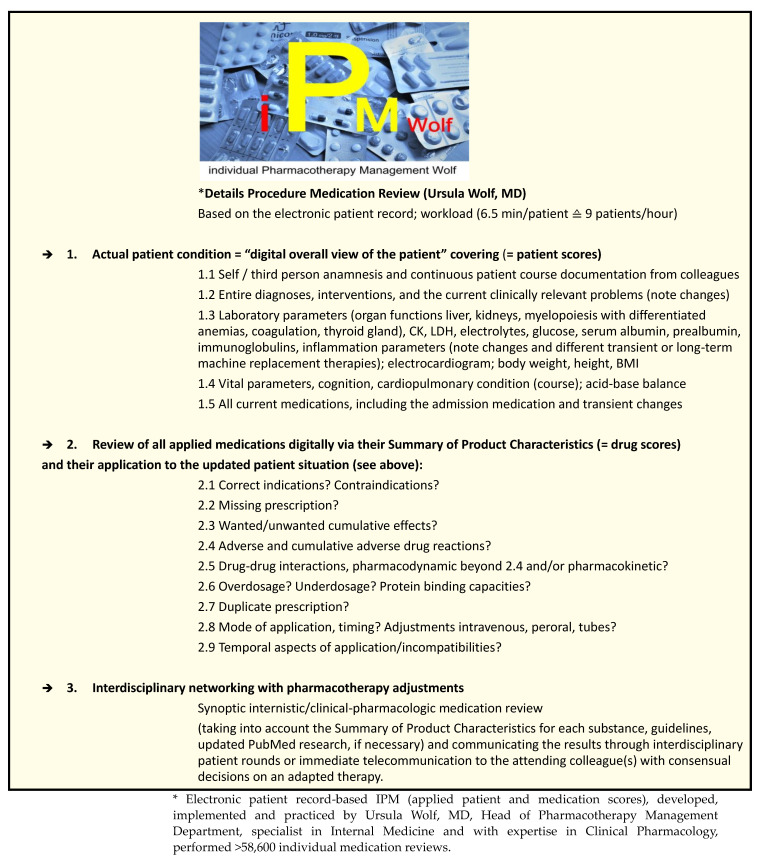
Comprehensive, reproducible IPM based on the electronic patient record.

Studying intensely the available highly updated electronic patient records provides the most comprehensive insights into the precise acute and chronic clinical patient condition as the essential digital basis for the entire variables addressed within the IPM concept to adapt each drug to the individual patient situation with reference to its ADRs, pharmacodynamic and pharmacokinetic DDIs, dosage, warnings, contraindications, correct application mode and timing (Figure 1).

### 2.4. Biomedical Statistics

Statistical analyses were performed with anonymous patient data using the statistical program SPSS version 23.0.0 for Microsoft Windows (SPSS Inc., Chicago, IL, USA). 

Descriptive data are presented with absolute and relative frequencies and mean, median, and standard deviation. We performed cross-tabulations to determine the relative frequency of potential association of IPM with bivariate redGFR and tested for independence or associations by using regression analyses and chi-square tests. Multiple logistic regression analysis was performed to examine the association between potentially associated factors and IPM.

## 3. Results

### 3.1. Study Groups’ Characteristics

**Figure 2:**
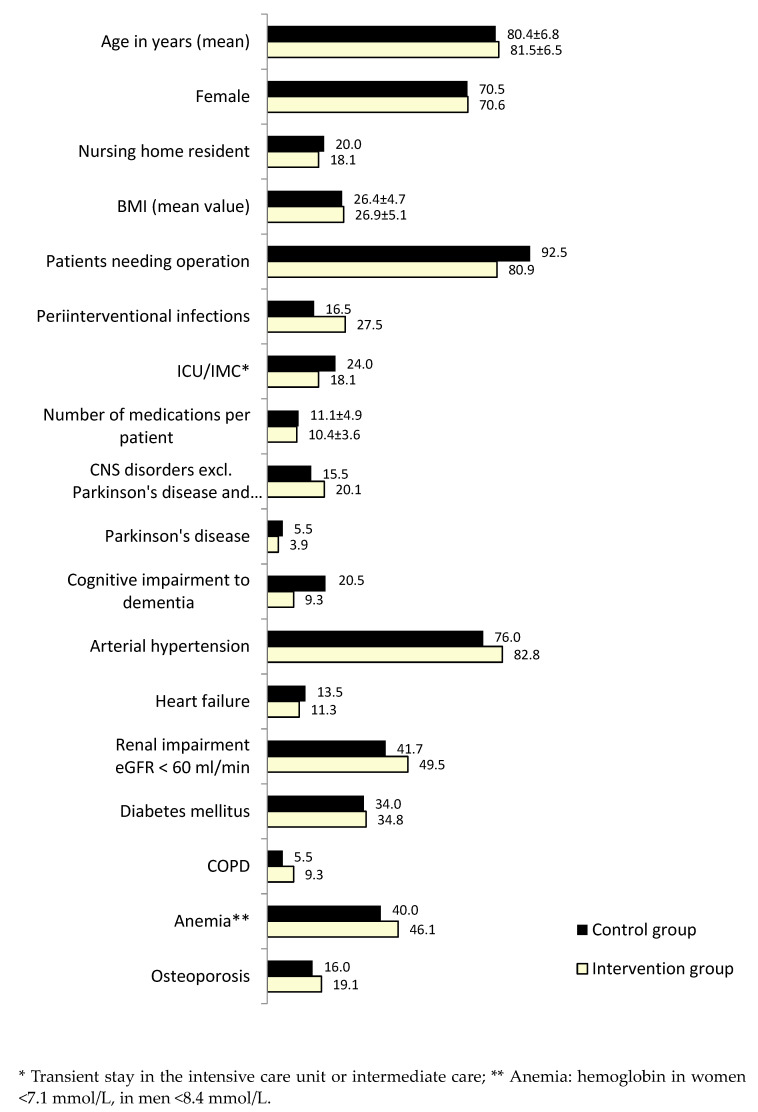
Baseline data and diagnoses accounted comparing CG and IG (prevalence percentages, except for age, BMI, and number of medications mean ± SD).

**Figure 3:**
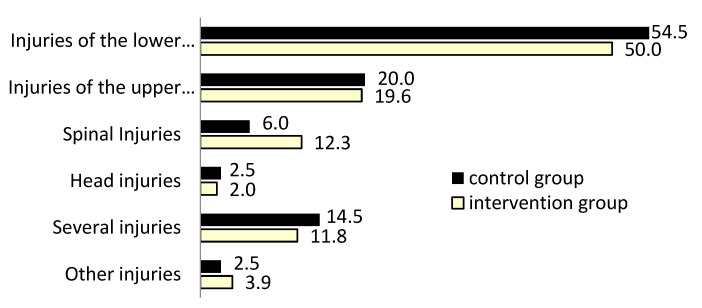
Injury pattern (%) of the lower and upper extremities and others in geriatric patients admitted to the traumatology department, comparing control and intervention group.

Despite ad random recruitment, the resulting IG and CG patient populations were found to be matched for age, gender, residency, BMI, CKD-relevant diagnoses (Figure 2), and injury patterns (Figure 3). Both patient groups were almost oldest-old ≥80 yrs of age, and most frequently, in 71%, women were affected by fall events and fractures demanding hospitalization. 

The two decisive chronic diseases with an impact on renal function showed a similar prevalence as for arterial hypertension 76% in CG versus 83% in IG, and diabetes mellitus 34% in CG versus 35% in IG. 14% in CG versus 11% in IG were diagnosed with heart failure (Figure 2). 

The high rate of anemia, defined as hemoglobin in women <7.1 mmol/L, in men <8.4 mmol/L with 40% (CG) and 46% (IG) was often associated with vitamin B12 deficiency as e.g., drug-induced metformin-treated diabetes patients or related to folic acid deficiency due to nutritional disorders, also prevalent in these elderly patients. A first glance at MCV from the small blood cell count already indicated these deficiencies via macrocytosis. Whether the overall elevated anemia rate in IG patients at hospital admission is related to the forthcoming increased anticoagulation with direct oral anticoagulants (DOACs) (Figure 6), remains to be investigated. From our experience, not figured, the rate of microcytic hypochromic anemia as an indicator of iron deficiency in this patient group has been observed to be slightly increased. Both types of anemia as well as renal anemia, aplastic, and others were differentiated, but are not displayed here. In this context, it is evident from the prescription rate that anticoagulant therapy by DOACs was increased 20-fold in IG without a parallel decrease in phenprocoumon prescribing, reflecting predominantly the increasingly stricter targeting of anticoagulation for nonvalvular atrial fibrillation (AF) besides treatment/prophylaxis of deep vein thrombosis and pulmonary embolism. Drug-induced anemias were targeted, e.g., vitamin B12 supplementation for metformin-induced macrocytic anemia, and all other types of anemia to exclude them in all elderly and eliminate anemia as a further risk factor for e.g., falls and dementia.

Osteoporosis diagnostic measures have been standardized by the traumatologists for these older trauma patients and contribute to the vitamin D supplementation presented (Figure 6). However, the final rate is not fully captured here because often treatment did not start until discharge after receipt of the entire diagnostic results e.g., from bone densitometry. The data even more reflect the low prescription rate of more commonly underprescribed vitamin D supplementation at admission and are probably in part a corresponding causal factor for the increased risk of fall-related manifest fractures, particularly in the predominantly female patients with postmenopausal osteoporosis. In this context, the adequate outpatient countermeasure of vitamin D supplementation in patients receiving long-term cortisone treatment or aromatase inhibitors was also frequently overlooked.

The indication for operative intervention was at high percentage in the elderly trauma patients, ranging from 80.9 (IG) to 92.5% (CG) in our study population. 

Antibiotics are known to increase the risk of renal injury, as do infections, such as urinary tract infections themselves. Although IG patients had an increased rate of various periinterventional infections, reflected by an identically higher rate of antibiotic therapy (16.5% in CG vs. 27.5% in IG) (Figure 6), IPM-IG patients did not develop redGFR during IPM. This may be related to the stringent IPM approach of fine-tuning antibiotic dosing to eGFR and close-meshed eGFR monitoring in predisposed CKD for further timely adjustment. If eGFR was not taken into account, overdose and increased nephrotoxicity of antimicrobial agents were alarming. Moreover, non-typical nephrotoxic agents had to be considered. In this context, we kept in mind, for example, that even the common combination piperacillin/tazobactam may be associated with delayed recovery of renal function compared to other beta-lactam antibacterial drugs (OR 1.7, 95% CI 1.18–2.43) and is a risk factor for acute kidney injury [26,27], especially in the late elderly [28]. This has been addressed by IPM through eGFR monitoring during treatment. 

It is particularly important to note that, because IPM was performed with interdisciplinary patient visits 2 weeks apart, especially in those 5 patients who developed redGFR in the IG before or markedly after IPM, redGFR was associated with antibiotic doses that were not matched to their individual renal function and eGFR follow-up, indicating that this clinical situation is a typical constellation for iatrogenic drug-induced risk of redGFR in elderly trauma patients, which requires even more frequent IPM to make timely corrections within these additional emerging unpredictable conditions.

The perceived need for usually very short postoperative transient intensive care in severe multimorbid and cardiovascular highest-risk elderly patients, represented by the referral rate to the ICU or IMC ward, was reduced by one-fourth from 24% to 18% in the IG. In contrast to 2 patients in the CG, none in the IG developed end-stage renal disease (ESRD) necessitating renal replacement therapy.

Primarily, 50% of patients in both groups were affected by trauma injuries to the lower extremity (Figure 3). Only the prevalence of spinal injuries was higher in the IG. Injuries to the upper extremity, head, or several concurrent injuries on admission were distributed almost equally.

Renal impairment, defined as eGFR <60 mL/min/1.73 m^2^ at hospital admission, was more prevalent in the IG, with 101 (49.5%) patients, than in the CG, with 83 (41.7%) patients. For the purposes of this study, we had to exclude one CG patient who lacked eGFR control. The distribution of differentiated stages CKD 3a to 5 is shown in Table 2 [18]. 4 patients in the CG and 6 patients in the IG were on chronic hemodialysis. In addition, transient hemodialysis was required postoperatively in 2 patients in the CG, whereas none in the IG. CKD was defined on the basis of eGFR values.

An important concerning finding was that the prevalence of CKD from the ICD-coded diagnoses in the patients’ discharge letters did not match the eGFRs determined, missing a high proportion of geriatric patients with impaired renal function: in the CG ICD-coded CKD 18.5% instead of 41.7% (=23.2% less), in the IG ICD-coded CKD 21.1% instead of 49.5% (=28.4% less). The discrepancy must be assumed to be even higher because the MDRD-GFR estimates only included CKD stages 3–5.

**Figure 4:**
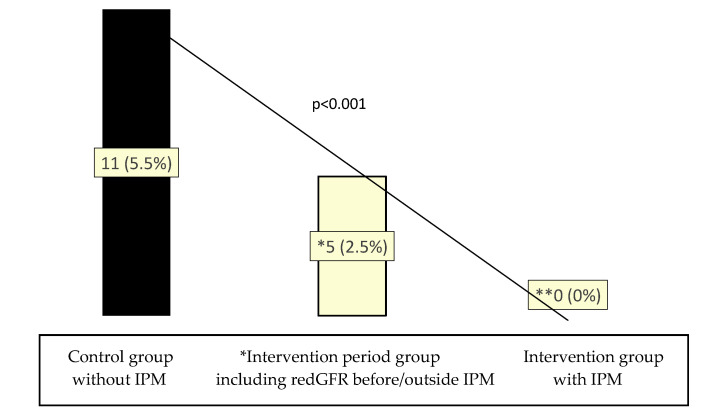
Comparing redGFR rate in the CG 11/199 (5.5%) with the IPM-visit IG 0/199, (0%) and trendline. * Due to the 14-day IPM ward rounds interval and blinded recruitment the intervention period group (204 patients) also included 5 patients who manifested redGFR before/outside IPM whereas for the IPM patients in context with the visit redGFR frequency reached zero. * There is an OR of 0.48 [95% CI 0.438–0.538], *p* < 0.001, already for the intervention group even including all patients with redGFR before/outside IPM. ** For the IG with IPM there was a complete absolute risk reduction by 5.5%, relative risk reduction 100%, NNT 18.

A decline in renal function was referred to focusing on the course of the eGFR values. Measurement of albuminuria as well as long-term monitoring of eGFR values for accurate calculation of possible progressive CKD stages were not applicable in the study in our geriatric traumatology ward due to the unavailability of contemporaneous urinalysis and relatively short hospital stay. A total of 11 patients (5.5%) developed redGFR within the CG. None of the patients from the IG period who received an IPM revealed redGFR in its context. However, because patient recruitment was blinded at all except for the inclusion criteria IPM period and age ≥70 years, and since the IPM ward rounds were conducted 14 daily only, we identified single patients who experienced redGFR already prior to IPM despite being registered in the IPM group (Figure 4). However, still in this entire IPM period group including even these patients outside the IPM, the number of redGFR was reduced to 2.5% (5 patients). This may be due to the introduction of standard operating procedures that resulted from the regular performance of IPM, such as fully reconfigured analgesic medication and constant attention to early dose adjustments for renal function and pharmacokinetic DDIs. Possibly, on-call physicians during the weekend or at night were not as familiar with this, leading to incorrect dosing of antibiotics in some cases.

### 3.2. Polypharmacy and IPM

**Figure 5:**
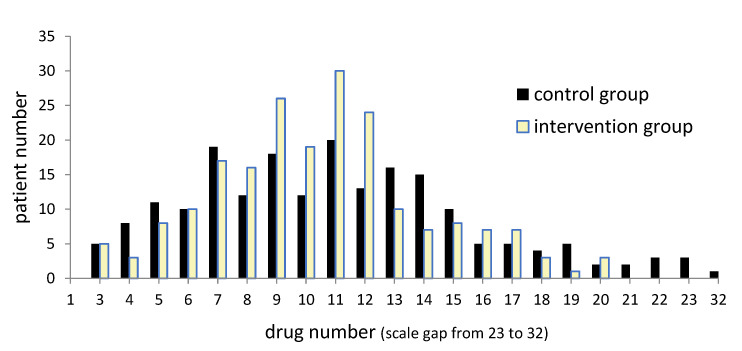
Number of drugs per hospitalized trauma patient comparing CG (11.04 ± 4.8; median 11) and IG (10.45 ± 3.58, median 10) with reference to the periinterventional entire medication list. All initiated despresciption of drugs, which require gradual dose reduction, e.g., antipsychotics, benzodiazepines or alpha blockers, are not reflected here because the perioperative stay in the trauma department was usually short, and thus medication withdrawal was not completed during the follow-up period at UKH.

The elderly patients at the trauma department were on an extensive spectrum of drugs due to their outpatient polypharmacy, mostly resulting from multimorbidity as arterial hypertension and cerebrocardiovascular diseases, heart failure, diabetes mellitus, COPD, dubious indications for psychotropic agents, urologics, and in addition transient medication in the perioperative course e.g., analgesics, antithrombotics, antibiotics. The challenge for IPM, therefore, is to be as all-encompassing and detailed as possible, since older adults often already suffer from concomitant deteriorated organs. On average, only marginally fewer medications were prescribed in the intervention group (10.45 medications/patient; ±SD 3.58, median 10) than in the control group (11.04 medications/patient; ±SD 4.8; median 11) (Figure 5). In particular, because further IPM foci for reducing fall risk and delirium in these geriatric trauma patients was also discontinuation of anticholinergics, benzodiazepines, antipsychotics, and alpha-blockers. They require gradual discontinuation, and because of the short hospital stay during the perioperative course and since the dosages and dose reductions are not included in this data collection, the resulting rate of final discontinuation by in-hospital initiated dose reduction cannot be displayed here.

**Figure 6:**
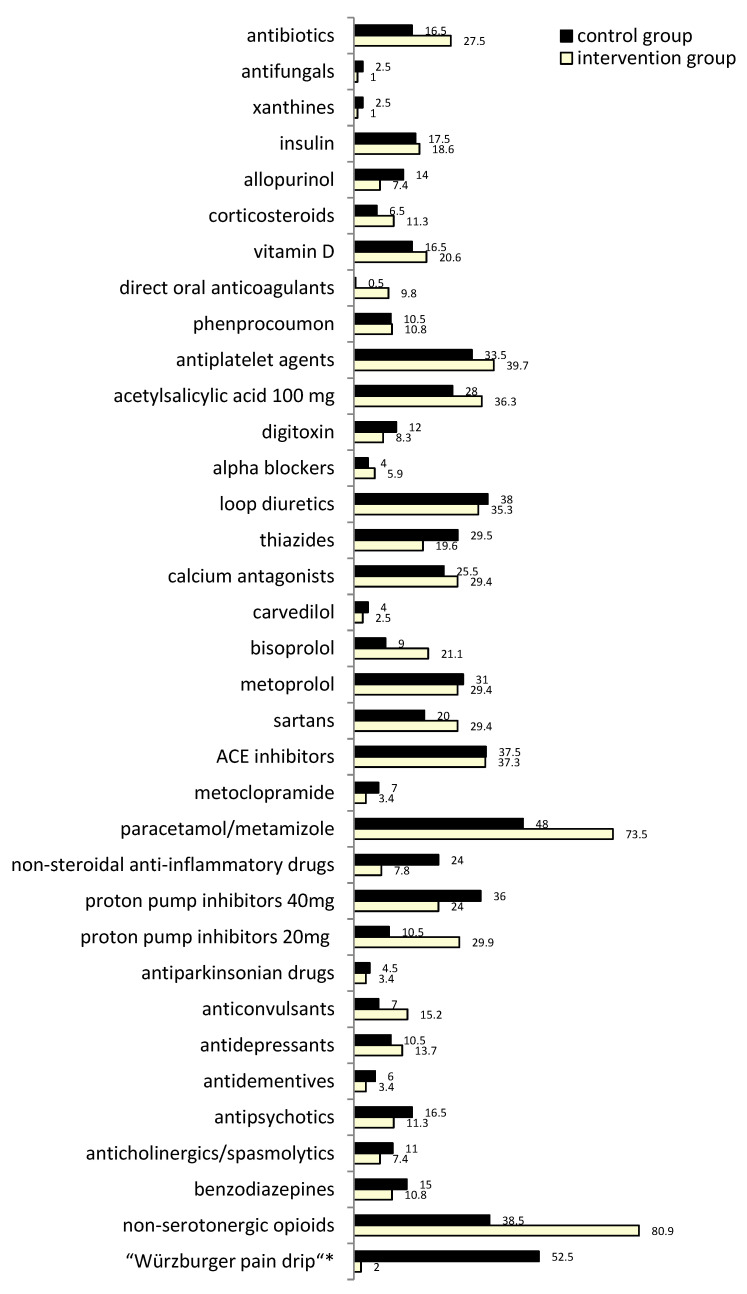
Prescription rates (%) of drugs and drug groups comparing CG and IG. Gradual discontinuations of, e.g., antipsychotics, benzodiazepines or alpha blockers are not figured here because most often not completed during the short hospital stay. * Combination of tramadol, metamizole, and metoclopramide—intravenously or partially orally.

The prevalence of the drugs and drug groups analyzed is shown in Figure 6. All IPM medication reviews were typically based on the respective current SmPC of each drug. The main IPM focus, as a synoptic view of internal medicine and clinical pharmacology, always referred to the analysis of indication and contraindication or missed indication, accurate dosing considering current renal and hepatic function, inhibition of metabolism/excretion with enhanced drug exposure, risk or manifestation of ADRs and pharmacokinetic and pharmacodynamic DDIs leading to cumulative ADRs, and correct use in terms of time intervals and mode of application (Figure 1).

In this regard, the overall IPM efforts are aimed primarily at drug and patient safety in terms of eliminating drug risks for cognitive impairment, falls, organ injury, inappropriate blood pressure, hydration, electrolyte imbalances, glucose, other metabolic and endocrinologic disorders, anemia, hypoxemia, and timely intervention for antibiotic-requiring infections.

To protect these hospitalized trauma patients, averaging >80 years of age, from further renal injury or even acute on chronic renal failure, often postprocedural or caused by hypovolemia due to concurrent illness, e.g., upper or lower respiratory tract infection, urinary tract infection, etc., throughout the perioperative course, a major aspect of IPM has always been to address both the nephrotoxic risks of a single drug and the cumulative risks from the patient’s drug list, paying particular attention to the corresponding ADRs and DDIs with regard to preexisting CKD, metabolic, endocrinologic, and cardiovascular risk factors, electrolyte and acid-base balance, and adequate and balanced hydration.

The targeted exclusion of any kidney deteriorating progress from, e.g., increased risk of nephrotoxicity in frequently preexisting renal impairment in elderly patients, was especially managed through these IPM-resulting measurements:

1. Dose fine-tuning of all drugs from the patient’s overall perioperative medication list adapted to current renal function and pharmacokinetic DDIs, not least with antibiotics.
2. Blood pressure optimization,
3. Targeted treatment of bradycardia, tachycardia, and arrhythmias,
4. Exclusion of hypo- and dehydration,
5. Avoidance of single and cumulative nephrotoxic risks from direct drug actions as, e.g., from NSAIDs mono or even coadministered with ACE-inhibitors or sartans,
6. Avoidance of single and cumulative indirect nephrotoxic risks from ADRs, e.g., from statins, and pharmacodynamic DDIs,
7. Early treatment of bacterial urinary tract infections,
8. Compensating for electrolyte and acid-base imbalances by timely targeted discontinuation of intensifying medications and, when compatible with respiratory capacity, bicarbonate use,
9. Following standard operating procedures for preventive measures in contrast to media administration.

Thus, referring to all dimensions of rhabdomyolysis from the widespread use of HMG-CoA reductase inhibitors, their ADRs, such as even cryptogenic chronic myositis and rhabdomyolysis, are often dose-dependent. The bioavailability of the statins atorvastatin, lovastatin, and simvastatin, e.g., when co-administered with strong CYP3A44 inhibiting agents such as, e.g., defined antimicrobials or antiarrhythmics like amiodarone, increases, as do their ADRs. In this context, pravastatin, rosuvastatin, and fluvastatin are more inert due to their different metabolic and elimination pathways. We had to adjust simvastatin when interacting with frequently coadministered CYP3A4- and/or P-glycoprotein-inibiting amlodipine. Further examples of consequent measurements were an eGFR-adapted dose of pravastatin to prevent the risk of chronically ongoing slight rhabdomyolysis from a twofold exposition of the drug and its metabolites [29]; the reduction in non-adjusted moxonidine [30] at risk of severe bradycardia, especially with concomitant administration of ß-blockers, therefore, caution when the drug is considered an “add-on” treatment option for hypertension in elderly patients [31]; and the adjustment of frequently overdosed uicostatic allopurinol, known to exhibit renal toxicity per se at an unadjusted overdose of 300 mg daily in patients with eGFR < 20 mL/min [32]. It turned out that all these aspects were not considered or neglected, and that these risks were predominantly already present in the outpatient medication list at hospital admission, for an unidentified duration.

To prevent further renal impairment or even acute kidney injury (AKI), an acute kidney failure, besides targeted drug cessation, as for, e.g., NSAIDs, which we reduced from 24 to 7.8%, or the early onset including timely dose adjustment of antibiotics, also the appropriate starting of bicarbonate in metabolic acidosis was IPM-recommended, always provided simultaneous monitoring of adequate respiratory capacity, partial pressure of carbon dioxide (pCO_2_) and acid-base balance. Separate individual cases of residual NSAID indications resulted from preventive measures for heterotopic ossification prophylaxis at certain predisposing sites of risk.

Clinically relevant hypotension with the inherent risk of renal hypoperfusion, as well as orthostasis, dizziness, and fall events or cognitive impairment and delirium often resulted from coadministration of antihypertensives including diuretics and antipsychotics and/or opioids. Dose reductions not additionally collected and presented here, or drug cessation, were necessarily initiated. We intentionally adhered to a slow deprescribing mode of alpha-blocking agents because of their risk of a rebound high blood pressure phenomenon, thus, these dose reductions are not covered in the figured medication prevalence rate (Figure 6). We also had to discontinue carvedilol e.g., when contraindicated in patients with COPD [33], and switched to cardioselective ß-blockers instead, e.g., bisoprolol. 

In addition to these ADRs and pharmacodynamic DDIs, there were also pharmacokinetic DDI risks that required, e.g., replacement of metoprolol due to CYP2D6-inhibited degradation with concomitant drugs such as melperone or various antidepressants, e.g., fluoxetine and paroxetine to a high extent, sertraline [34] and citalopram to a moderate extent, the latter also requiring renal dose adjustment, which was usually already overlooked in the outpatient medication. The primary IPM intention was to gradually discontinue antipsychotics, prior to metoprolol replacement if possible. When this was not applicable, the cardioselective ß-blocker bisoprolol was administered alternatively because it is inert with respect to these pharmacokinetic metabolic DDIs with cumulative risks. To exclude any CYP2D6-associated pharmacokinetic risk in this context, the bisoprolol prescription rate accordingly increased from 9% in the CG to 21% in the IG (Figure 6).

Exclusion of cumulative risk from drug-related manifest severe hyponatremia, potentially resulting from coadministration of diuretics e.g., hydrochlorothiazides (HCT), and spironolactone, ACE-inhibitors or sartans, and a broad spectrum of antidepressants as selective serotonin reuptake inhibitors (SSRIs) or selective serotonin-norepinephrine reuptake inhibitors (SSNRIs) or mirtazapine was primarily targeted by cessation of HCT and spironolactone or gradual deprescription of antidepressants when applicable. Sometimes, differential diagnoses such as a syndrome of inappropriate antidiuretic hormone secretion (SIADH) required additional measurements of serum and urine osmolalities and check for elevated urinary sodium at normal dietary salt and water intake. Versus hyperkalemia, one focus was on avoiding a pairing of aldosterone antagonists and sartans or ACE inhibitors. In addition, IPM also accounted for the need for dose adjustment to renal function within these drug groups, e.g., ramipril [35] or enalapril [36].

The always time-limited combination of loop diuretics and HCT, preferentially xipamide, as IPM-targeted in individual cases for benefit from transient sequential nephron blockade, was an intervention that is also known to further improve diastolic function in patients with resistant hypertension [37]. 

In patients receiving bisphosphonates, prescribed e.g., for postmenopausal osteoporosis, we had to interrupt intake in the presence of hypocalcemia deserving calcium substitution, as this is a contraindication for administration of these drugs. We furthermore identified single cases with specific risk in bisphosphonate use in advanced renal impairment requiring discontinuation, e.g., alendronate is not recommended in patients with eGFR < 35 mL/min because of insufficient experience [38].

In addition to the already standardized perioperative pause and interruption with iodinated contrast media and nephrotoxic drugs or renal hypoperfusion, the precise adjustment of metformin to avoid lactic acidosis with risk of acute kidney injury or its discontinuation in severely impaired renal function with eGFR < 30 mL/min was also addressed [39], although some data confirm potential benefits from cell-protective effects in ischemia/reperfusion-related injury by reducing reactive oxygen radicals via activation of adenosine monophosphate kinase that may outweigh the risks of lactic acidosis [40,41,42,43].

The adequate e-GFR-adjusted dose of metamizole, still a favorite postoperative analgesic in Germany to avoid high opioid loads, was an IPM focus, too [44,45].

Because of their anticholinergic properties, we also withdrew various inadequate spasmolytics for the pharmacologic treatment of overactive bladder. The anticholinergic therapy using muscarinic receptor antagonists, and tertiary or quaternary amines often revealed overdosage e.g., trospium chloride in renal impairment. It is mostly overlooked that trospium chloride is excreted mainly by the kidneys, thus significant increases in plasma levels have to be expected from observations in patients with moderate and severe renal impairment. Therefore, this group of patients, even with mild renal impairment, should be treated with caution according to the SmPC [46]. Since the positive charge makes it a hydrophilic drug, it is unlikely to cross the blood-brain barrier and the potential for central ADRs is low compared to other anticholinergics such as oxybutynin chloride, which as a tertiary amine, and with its high lipophilicity, neutral charge, and low molecular weight, can more easily penetrate the blood-brain barrier than the quaternary amine trospium chloride [47] and was thus consequently withdrawn contributing to an immediate anticholinergics/spasmolytics reduction from 11 to 7.4% (Figure 6).

Regarding the protein binding aspect, it is important to note that almost all elderly trauma patients, irrespective of their renal function, had subnormal serum albumin and prealbumin levels at admission, as evidenced in the 12-year IPM routine. This is an important aspect to consider as it affects the delivery and bioavailability of drugs with high protein binding, which is further exacerbated by their additional binding competition. Accordingly, extra focus has been directed to a supplemented high-protein nutrition in these elderly patients from the earliest, providing regular hepatic function.

As illustrated in Figure 6, the outlined IPM strategies resulted in a profoundly different analgesic drug regimen with an overall significant reduction in NSAIDs in the IG (*p* < 0.001), alternatively in an increased prescribing of non-serotonergic opioids (*p* < 0.001) as hydromorphone instead of the “Würzburger pain drip” which includes serotonergic tramadol, plus metamizole and metoclopramide (*p* < 0.001). IPM focussed on the intentional reduction in proton pump inhibitor (PPI) dosage to a preventative dose level of 20 mg instead of the almost typically overdosed 40 mg daily. As further significant consequences of the IPM procedure compared to CG, the IG differed by a significant reduction in HCT (*p* = 0.028), most frequently because of associated hyponatremia or dehydration and manifest hypotension; and antibiotics were administered significantly more often (*p* = 0.004) with focus on fine-tuned dosage.

Due to the brevity of acute inpatient trauma care, the implications of the overall subsequent deprescribing processes cannot be reflected in this figure because additional data collection of all individual dosing regimens for each drug administered was not performed. Our very ambitious deprescribing measures, particularly of antipsychotics, serotonergic opioids, and benzodiazepines in terms of preventing delirium and cognitive decline as well as fall events, required a gradual downshift rather than abrupt discontinuation, and the data shown cannot reflect the deprescribing trend because the inpatient stay in traumatology usually means only a short period of treatment and postoperative patient observation and mobilization. For this reason, the physicians providing further care to the trauma patients were informed of the IPM results and the pharmacotherapy adjustments initiated by the physician’s discharge letter and instructed to continue tapering for final discontinuation.

Despite all IPM efforts, some risk of manifestation of redGFR due to unconscious renal dysfunction and inadequately adjusted antibiotic dosing persisted independently. On-duty physicians from outside the department who were not involved in IPM and who administered these drugs for acute illnesses outside of regular patient rounds, such as on weekends, appeared to be involved. This was predominantly the case in the 5 patients with redGFR in the IG outside the IPM. This serious problem of unrecognized renal impairment is also reflected in the list of ICD-coded diagnoses in the patients’ discharge letters. The ICD-coded diagnosis of CKD was only 18.5% compared to 41.7% according to eGFR in CG and 21.1% compared to 49.5% in IG. Obviously, the focus here is much more on avoiding surgical risks and complications and the diagnoses that the patient brought from the history, although these anamnestic diagnoses may already be outdated for the current condition. To further verify and diagnose new unknown diseases such as CKD, probably only minor attention apart from the immediate care of the acute intervention event remains unless the patients are transferred analogously to the appropriate responsible internistic disciplines after consultation.

Although these almost oldest-old patients, with an average age of ≥ 80 years, suffered from multimorbidity, and thus polypharmacy was unavoidable, we hereby demonstrate that despite high numbers of medication, necessarily even enhanced by analgesics, antibiotics, and antithrombotics during the perioperative course, as shown here, the IPM associated with complete elimination of redGFR was highly effective in these challenging conditions, as it aims at a most comprehensive and individualized drug and patient safety. 

### 3.3. Analytic Statistics on Associations of IPM and redGFR 

In terms of potential confounders such as age, gender, BMI, arterial hypertension, diabetes mellitus, and injury pattern, the group-matched study on 403 trauma patients, comparing the non-IPM-control group (CG) with the IPM-intervention group (IG), revealed a complete absolute risk reduction of redGFR by 5.5% (11 of 199 CG patients) to 0% (0 of 199 patients) in the IPM-IG, meaning a relative risk reduction of 100% and NNT 18. This indicates immediate high effectiveness of IPM improving outcomes, since no patient was found to have worsening renal function by the defined redGFR in the temporal relation to the IPM visit. 

**Figure 7:**
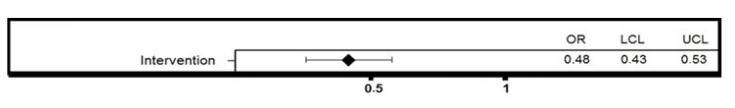
Measurement of an even potentially superimposed IPM effect. Association of IPM with redGFR including the IG patients presenting redGFR before or outside the IPM visit, *p* < 0.001.

According to the contingence analysis on a superimposed effect on the total patient group of the intervention period, regardless of whether IPM had already been conducted or not, redGFR was also significantly lower with a relative risk reduction = 0.55; 55%). In this studied entire intervention group, including some patients still awaiting IPM, the risk of redGFR was 2.5% (5 of 204 patients). A follow-up of these 5 patients revealed, that 3 patients had already developed redGFR before the IPM visit because of its 14-day intervals, and 2 others experienced redGFR at a >1-week post visit distance in the context of a new-onset infection warranting antibiotics that required dose adjustment. Nevertheless, the resulting measure of association of a potentially superimposed IPM effect with redGFR including these patients without or outside the temporal association with the IPM visit also yielded an OR of 0.48 [95% CI 0.438–0.538] with high significance at *p* < 0.001 (Figure 7). 

In total, 16 patients of the entire CG and IG developed redGFR: 1.4% (3 of 216 patients) with admission eGFR ≥ 60 mL/min/1.73 m^2^ (with normal renal function, CKD 1 or CKD 2); 13 patients with admission eGFR < 60 mL/min/1.73 m^2^, including 7.1% (6 of 79 patients) with CKD 3a, 10.5% (6 of 51 patients) with CKD 3b, and 3.3% (1 of 29 patients) with CKD 4.

We also analyzed whether an application of the BIS-1 formula [14] introduced in 2012 as an equation to estimate renal function in patients ≥ 70 years of age, according to our study population, revealed different eGFR values compared to the MDRD formula, probably relevant in terms of altered CKD distribution patterns and predictability of redGFR. BIS-1 values were calculated for each patient per online tool. As shown in Table 3, the mean values of BIS-1 and eGFR were relatively close in the lower range for eGFR 0–29.9 mL/min, whereas at higher eGFR values of 30–59.99 mL/min, the mean eGFR and BIS-1 values differed more clearly by 4.54 mL/min, which may highlight the relevance and possibly higher sensitivity of using the BIS-1 formula to differentiate in CKD stages above 4 such as CKD 3a and 3b and to avoid overestimation of eGFR.

In the context of this study, our recommendation to adequately assess and further differentiate the renal function ≥ 60 mL/min/1.73 m^2^ at UKH led to the introduction of the eGFR CKD-EPI instead of the MDRD formula in 2016. Thus, only in the later course of the IPM intervention phase, a small proportion of patients were assessed by this method. However, since the majority of the IG was estimated analogously to the entire CG via eGFR MDRD with the unification of all GFR values ≥60 mL/min/1.73 m^2^, we could not conduct a BIS-1 comparison for CKD stages 1 and 2 additionally. 

The application of BIS-1 indicates that the number of risk patients for redGFR from the CKD 3b stage would even be enhanced, shifting more patients from CKD 3a to CKD 3b. This would be of clinical relevance, as it was the patient group most prone to red GFR (10.5%), who therefore require particular vigilance and closer monitoring of eGFR. 

For patients with an IPM and an absolute risk reduction of eGFR of 5.5% (11 of 199 in CG versus 0 of 199 in IPM-IG)), a relative risk reduction of 100%, and a number needed to treat of NNT = 18, the latter cannot even be further reduced because it is impacted by the overall low incidence of the very strong outcome redGFR in the CG already.

Contingency analysis for the entire patient study population on the association of redGFR (*n* = 16) with the clinically relevant variables selected from the results of the cross-tabulation calculations, adding established variables from literature data, was performed for age, gender, CKD, diabetes mellitus, arterial hypertension, NSAIDs, contrast media, allopurinol, Würzburger pain drip, antibiotic-requiring infections, bacteriuria, loop diuretics, thiazides, ACE-inhibitors, and polypharmacy (number of prescribed drugs). We applied a chi-square test and the resulting *p*-values including Fisher’s exact test for correction because of small samples and low numbers of expected frequencies revealed significant associations of redGFR with allopurinol (*n* = 6) *p* = 0.002, bacteriuria (*n* = 2) *p* = 0.007, loop diuretics (*n* = 11) *p* = 0.014, eGFR ≥60mL/min (including patients with normal kidney function, CKD 1 or CKD 2) (*n* = 3) *p* = 0.003, eGFR <60mL/min (*n* = 13) *p* = 0.004, and CKD 3b (*n* = 6) *p* = 0.016. The limited strength of the results due to low case numbers has to be considered. Additional potential confounders such as age, gender, diabetes mellitus, arterial hypertension, number of medications, and contrast media did not reveal a significant association with redGFR; again, a limited power has to be respected due to the very short periinterventional follow-up period as well as the small number of concerned patients.

Multiple binary logistic regression analysis was performed to estimate the probability of an IPM-associated reduction in the dependent variable redGFR with respect to the simultaneous effects of multiple explanatory independent variables. Besides the model with our own verified variables we considered established risk factors for acute kidney injury: age, gender, arterial hypertension, diabetes mellitus, heart failure, CKD stages with eGFR <60 mL/min/1.73 m^2^, NSAIDs, ß-blockers, loop diuretics, thiazides, ACE inhibitors, paracetamol (acetaminophen), PPI, contrast media. The regression coefficient of the IPM (−1.437) was found to be significant (Wald(1) = 4.390, *p* = 0.036) even including all patients of the IG before/week after IPM, indicating IPM was associated with a superimposed reducing significant impact on redGFR with an effect outside the individual IPM visit, e.g., via optimized standard operating procedures on the part of the regular IPM in the intervention period as demonstrated for our completely IPM-altered analgesic treatment. In this model, the relative probability of redGFR was significantly reduced with OR = 0.238 [95% CI 0.06–0.91] for this entire IPM setting, confirming the assumption of a superimposed effect of IPM. The relative probability of a redGFR was reduced by 76.2% during the intervention period, even including patients before/beyond the 14 daily IPM visits. The regression coefficients of the examined independent variables of the multiple logistic regression were significant, indicating a relationship, between redGFR and the following independent variables: CKD <60 mL/min/1.73 m^2^ regression coefficient 2.893, *p* = 0.001; loop diuretics 1.397, *p* = 0.036; HCT −2.575, *p* = 0.023; IPM −1.437, *p* = 0.036. Again, there were no further associations with age within the already nearly homogeneous group of elderly to oldest patients, nor with gender, arterial hypertension, diabetes mellitus, heart failure, ACE inhibitors, ß-blockers, or contrast media.

## 4. Discussion

### 4.1. IPM Effectiveness and Evaluated Outcome

The profound personal and socioeconomic implications of an increasing prevalence of renal dysfunction with all its accompanying cerebrocardiovascular comorbidities in an accelerating aging population urgently require strategies for the reliable prevention of CKD and acute kidney injury [48,49]. Our study focuses on a short-term course in the most typical susceptible elderly patient population undergoing periinterventional risks. To our knowledge, there are no study data on a similarly documented efficacy of preventing iatrogenic drug-induced renal injury, although not only by single drugs but exacerbated and cumulative in polypharmacy, the problem is known to be ubiquitous and increasing, demanding to be addressed more consequently and adequately [50]. Our comprehensive IPM accounting for the holistic patient condition from a synoptic internal medicine and clinical pharmacology perspective is associated with the potent prevention of renal impairment avoiding SmPCs-specified drug risks in polypharmacy through addressing the ADRs, overdoses, pharmacodynamic and pharmacokinetic DDIs, contraindications, and missing prescriptions. Referring to all these aspects in relation to the acute patient situation and his organ conditions, the vulnerable geriatric kidneys are accordingly guaranteed to be wrapped in absorbent cotton and treated with great sensitivity. The IPM internist has applied her decades of kidney transplant experience to the treatment of similarly vulnerable kidneys of geriatric and intensive care patients analogously in this conceptual IPM. Their often pre-impaired kidneys [51,52,53,54] pose an increased risk of iatrogenic renal injury from polypharmacy particularly. The IPM procedure with the correspondingly designed IPM patient scores and drug scores is as detailed and individualized as possible. This may be the main reason and explanation for its high effectiveness. Partly helpful tools for the prevention of polypharmacy risks are available [55,56,57,58,59,60]. However, literature data on the results of medication reviews [61,62] do not reach the IPM-associated effectiveness. They do not cover the entire acute patient situation in terms of the most comprehensive electronic patient data selection possible. 

The clinically relevant outcome redGFR ≥ 20 mL/min/1.73 m^2^ is strong to intentionally capture the more severe degrees of renal decline since less pronounced variations of eGFR around 10 mL/min throughout the perioperative might be of minor relevance. Especially in the perioperative setting, a redGFR around 10 mL/min only may result from a variety of multifactorial and often transient causes, such as anesthetic and analgesic drugs and acute surgical effects, hydration status, blood pressure drop, bleeding issues, or an infectious course. In consequence, our focus on a more robust and powerful renal function parameter was to enable the analysis of a significant IPM effect more accurately. Acute kidney injury (AKI), besides urine volume, is determined by creatinine levels [63]. However, for the geriatric patient population, serum creatinine is known to be less suitable and not a reliable marker for renal function compared to eGFR [19,64]. This seems somewhat inappropriate and inconsequential for accurately determining further renal impairment in the increasingly elderly patient population. Yet, creatinine use may be at least more suitable in the advanced CKD stages 3b to 4, where only a narrow GFR interval remains left to identify additional acute impairment. While the relationship between serum creatinine and GFR remains a reciprocal function, the estimation of GFR via creatinine-based equations accounts for other variables such as age, gender, and ethnicity. Of these, age-related decreases in renal function should be a special aspect to consider in the CKD definition [64]. We could not retrospectively collect additional AKI measures, such as changes in urine volume, since they are not routinely assessed in the trauma patient population. Because of the high value of the defined redGFR, its prevalence was not as pronounced even in the CG. The traumatologists were already used to monitor GFR before the IPM intervention, and adequate hydration in the elderly has always been a major focus of care. The predominant association of redGFR with impaired renal function was found in CKD >2 stages, especially in CKD3b. However, a significant risk of redGFR was also identified in patients with GFR > 60 mL/min/1.73 m^2^, although here the number of patients affected and thus its statistical power was considerably lower. 

### 4.2. CKD and eGFR Estimation Aspects

According to the 2012 Kidney Disease: Improving Global Outcomes (KDIGO) clinical practice guideline for the evaluation and management of CKD, the eGFR category is a risk predicting variable for the outcome of CKD to identify, apart from the cause of CKD, albuminuria category, other risk factors, and comorbid conditions [65]. The estimation of GFR in the study population was by the MDRD formula. The 2009 CKD-EPI equation introduced in the UKH at the end of our study has been shown to rate fewer adults having CKD, but more correctly categorize risk for mortality and ESRD (end-stage renal disease) compared to the MDRD study equation [23]. Since reduced muscle mass and decreased muscle activity, typically seen in elderly people, as well as low protein intake or severe malnutrition, make serum creatinine a non-suitable marker for kidney function, that is why eGFR has to be estimated especially in the elderly and eGFR also became the definition parameter for CKD. Yet, on the other hand, AKI is left to be determined by an increase in creatinine furthermore besides reduced urine flow. At the time of this study, there were no routinely measured biomarkers such as cystatin C levels available at the UKH. Serum cystatin C, as produced by all nucleated cells and filtered in the glomerulus, makes an estimation of renal function independent of origin and gender. It has been established as an early and more accurate biomarker for CKD to be used especially in patients in whom creatinine is an inadequate marker. The KDIGO guidelines suggest “measuring cystatin C in adults with eGFR 45–59 mL/min/1.73 m^2^ who do not have markers of kidney damage if confirmation of CKD is required.” [65,66]. Principally, each documented eGFR value must be considered with respect to its underlying assessment, varying accordingly, especially in older adults [67,68,69,70]. Currently, besides the new creatinine- and cystatin C-based equations to estimate GFR without race from the CKD Epidemiology Collaboration (CKD-EPI), evaluated to be more accurately than new creatinine-equations [71], furthermore the European Kidney Function Consortium (EKFC) has developed race- and sex-independent cystatin C-based equation for estimating glomerular filtration rate (GFR) by using a scaling factor for cystatin C, which appears to be more exact than the CKD-EPI cystatin C equation. The scaling factor applied was based on analyses showing that cystatin C levels did not differ between white and black patients of the same age, sex, body mass index, and measured GFR. They applied the creatinine-based equation (EKFC eGFRcr) to estimate GFR with this rescaled serum creatinine level, in which the serum creatinine level is divided by the median serum creatinine level among healthy persons to control for variation related to differences in age, sex, or race, and a cystatin C-based equation to estimate GFR without the inclusion of race and sex. The corresponding recently published comparative study on calculating GFR indicates that this current calculation, EKFC eGFRcr, based on creatinine concentration, performed equally well as the cystatin C use and referring to cohorts from Europe, the US, and Africa, this equation was shown to improve the accuracy of GFR assessment over that of the commonly used equations [72]. 

### 4.3. CKD Unawareness and Associated Risks

To move forward in the management of CKD, we should not only provide recommendations for specific drugs and lists of typical nephrotoxic agents to be avoided. Moreover, we have to advocate an IPM for the entire drug list, accounting for the individual patient’s overall clinical condition while evaluating indications, contraindications, adverse events, DDIs, and dosages.

It is an alarming result that possibly not only in surgical disciplines but also in outpatient care renal impairment is not always given the attention it urgently requires, especially in vulnerable geriatric patients often with preexisting renal impaired conditions. For the operative emergency setting, the main focus seems to be on avoiding surgical risks and complications and on secondary diagnoses that the patient has brought from the medical history. These diagnoses may already be outdated with gaps at the time of hospital admission. The unawareness of CKD even in advanced stages has been documented even more pronounced in various countries and medical disciplines [73,74,75,76]. Worldwide, only 6% of the general population and 10% of the high-risk population are reported to be aware of their CKD status, despite increasing CKD prevalence [75]. From our evidence, there is a risk that discharge letters might not always code all concomitant secondary diagnoses in addition to the actual predominant surgical one. We were told that surgical epicrises are frequently written all at once in retrospect just before hospital discharge, unlike the frequently continuous internistic course documentation. This might be one explanation, more than that it should be an intentionally short discharge letter. Our unexpectedly high degree of >50% undiagnosed CKD in stages 3–5 is critical for the patient. It may even affect the outpatient after discharge, in case the general practitioner providing further treatment relies on the listed diagnoses unless he knows the patient’s renal function himself from his own outpatient eGFR controls. The risk of unawareness of CKD among physicians and patients is increased by two additional circumstances: (1) CKD usually stays asymptomatic over a long time, being therefore diagnosed often at advanced stages, and (2) Nonrecognition will be amplified arising from the controversies about whether a decline in renal function with age should be considered more a normal physiologic aging process than a disease [77]. Categorizing it as normal would further enhance all our demonstrated risk from unawareness with its associated inappropriate non-adjusted dosing and contraindicated drug therapies. Our results advocate the opposite: CKD should be classified as a riskful and frequent disease in the elderly, demanding high attention and follow-up because of the multiple inherent medication consequences and CKD-associated cerebrocardiovascular diseases, increased hospitalizations, cognitive impairment, and premature mortality [78]. The health economic costs of CKD are especially related to the concomitant diseases of CKD [78], besides the extraordinarily high costs of ESRD necessitating renal replacement therapy by dialysis or renal transplantation. Thus, it is important to diagnose CKD at the earliest stage and to prevent its progression by all available means, e.g., by obligatory IPM, in order to (1) save future health care costs, and (2) ensure patient safety through drug safety by eliminating all kind of iatrogenic mediation-induced kidney injury.

### 4.4. Patients, IPM Focuses in Polypharmacy, Renal Risks and Prevention

A broad spectrum of renally eliminated drugs already requires dose adjustment in advanced CKD, on the one hand. The other risk arises additionally from the nephrotoxic ADR potentials of further commonly prescribed drugs, which are typically amplified with dosage or by unrecognized pharmacodynamic or pharmacokinetic DDIs. Correspondingly, the study identified manifest redGFR from overdosed antibiotics, such as in patients before or outside the IPM visit. This classic clinical risk situation for redGFR indicates a need for IPM to be performed even more frequently than every 14 days. There is a high fluctuation of prescribing physicians, particularly in the staffing of night and weekend duty physicians, often involved in acute antibiotic intervention and obviously not always aware of the patient’s renal impairment. The findings challenge an obligatory up-to-date eGFR documentation with at least every inpatient and outpatient medication list to promote and ultimately ensure appropriate dosing, especially in renally pre-impaired elderly patients in the presence of polypharmacy. Antibiotics and even additional DDIs pose a significant risk of further renal injury [10,79,80,81]. 

The higher rate of older women compared to men is not completely congruent with the population statistics for this age group. According to the German Federal Statistical Office, the gender distribution in 2021, e.g., among those aged 80–84 was: 41.9% men and 58.1% women, and among adults ≥ 85: 34.6% men and 65.4 women [82]. As the study refers to trauma patients, our even more elevated prevalence in women might be related to the increased trauma and fracture risk, not only in the elderly [83,84], but due to postmenopausal female osteoporosis. One in two postmenopausal women is estimated to suffer an osteoporosis-related fracture during her remaining lifetime, compared to one in five men [85].

Alongside the desirable efforts to deprescribe, some degree of polypharmacy often remains unavoidable in the case of multimorbidity, especially in old age. As underlined by our study results, this makes it all the more important to focus on optimizing this multimedication process, particularly for the age-related pre-impaired drug degrading or eliminating organs [86] and perioperatively further afflicted vulnerable patients. 

Our focus comprised the entire medication list, not only drugs known for their nephrotoxicity such as NSAIDs, aminoglycosides, etc. The identified association of redGFR with loop diuretics and allopurinol has to be considered with caution due to the small numbers and the very short follow-up period. It does not reflect drug injury that may result from long-term treatment or prolonged inappropriate dosing. However, kidney injury by diuretics resulting in AKI is well established and documented, with pathological details revealing vacuolar degeneration of tubular epithelial cells as a common lesion induced by diuretics [87]. In this context, age was a predictive factor for incomplete recovery and all-cause mortality. Clinical and pathological changes were more severe with high doses of furosemide, probably indicating dose-dependent injury [87]. For the effects of allopurinol on CKD, study results from the literature remain controversial. Hyperuricemia, not only linked to inflammation but also to the progression of renal and cardiovascular disease, was effectively treated with allopurinol with a beneficial effect on all of these conditions [88]. A recently published large randomized controlled trial (RCT) in the UK in patients ≥60 years of age did not show a difference in the primary outcome of non-fatal myocardial infarction, non-fatal stroke, or cardiovascular death between participants randomized to allopurinol therapy and usual care [89]. Yet, for the patients treated, the mean serum uric acid concentration at baseline was relatively low and the association between allopurinol-induced changes in serum uric acid concentrations and outcomes was not confirmed [89,90]. From our IPM insights for the serum uric acid-lowering drugs, primarily indicated in patients with symptomatic hyperuricemia, allopurinol often failed to be dose-adjustment in advanced CKD. Similar to febuxostat, known to have 2-4-fold increased exposure in severe renal disease with an eGFR < 30 mL/min requiring withdrawal because of insufficient safety yet, and it is not recommended in organ transplanted patients or in patients with ischemic heart disease or decompensated heart failure [91], aspects that are also widely ignored according to our IPM analyses. Despite broad experimental and epidemiological evidence for hyperuricemia as a CKD risk factor, there is in summary insufficient evidence for the therapeutic effect of allopurinol [92]. Accordingly, the KDIGO 2012 Clinical Practice Guideline for the Evaluation and Management of Chronic Kidney Disease states: “Hyperuricemia 3.1.20: There is insufficient evidence to support or refute the use of agents to lower serum uric acid concentrations in people with CKD and either symptomatic or asymptomatic hyperuricemia in order to delay progression of CKD. (Not Graded)” [65].

Already in 1991, drug complications were found to be the most common type of adverse event in hospitalized patients, accounting for 19%, calling for better medical knowledge and prevention of treatment errors, as well as overcoming a high level of negligence [93]. A significant prevalence of ADRs was also found in hospitalized patients in internal medicine departments; changes in renal function during hospitalization and DDIs appeared as important risk factors for ADRs [94]. Such conditions actually contribute to a mutually reinforcing spiral of kidney injury.

We identified an association of bacteriuria with redGFR. As known for CKD, there is an elevated risk of bacteriuria and urinary tract infections. This is partly due to CKD-associated metabolic and immunologic deterioration in terms of enhanced apoptosis of lymphocytes, increased levels of tumor necrosis factor α and interleukin-6 (IL-6), which depress neutrophil function, and enhanced levels of uremic toxins such as p-cresyl sulfate and indoxyl sulfate, which alter leukocyte adherence and migration at sites of injury. Furthermore, urinary tract infections precipitate worsening of renal function, especially in stages G3-G5 of CKD [95]. Not only is an elevated IL-6 level a consequence of CKD itself, but it also acts as a progression inducer for CKD, and IL-6 simultaneously triggers CKD-related chronic vascular disease by promoting atherosclerosis, for example [96]. Advanced age and impaired baseline renal function besides diabetes mellitus, upper urinary tract infection, and afebrile status, have been identified as risk factors for the development of AKI in patients with urinary tract infection [97].

The IPM measure resulted in reduced HCT therapy. However, we intentionally combined it temporarily with loop diuretics to improve diuresis in critically impaired conditions. This probably may explain the obviously positive effect of HCT with redGFR although with low power numbers. HCT is known to enhance the effect of loop diuretics even in more advanced CKD by sequential nephron blockade [98].

We have systematically reduced the almost classically overdosed and most frequently non-indicated PPI, the predominant pantoprazole from 40 mg to the prophylactic dose of 20 mg, in line with the gradual deprescription required in this context. Evidence has accumulated that PPI use is associated with an increased risk of CKD disease regarding the onset of CKD, progression of CKD, and renal failure [99]. Studies have consistently described a graded increase in risk with higher doses and longer duration of PPI therapy [100]. Accordingly, associations between PPI use and the risk of acute interstitial nephritis (AIN) and AKI have been reported in numerous studies, particularly among hospitalizations. Besides, a direct pathway of indolent chronic kidney injury has been assumed [100]. A demonstrated time association between exposure to PPIs and the occurrence of AIN is likely to strengthen a causal relationship [101]. It is of clinical relevance to know that PPI use is associated with an increased risk of chronic kidney injury, even in the absence of AKI. Thus, the authors warn, that, relying on AKI as a precautionary sign is not sufficient to reduce the risk of CKD in PPI users [101].

The “triple whammy” in primary care, the concomitant use of a triple therapy combination of ACE inhibitors or ARBs plus diuretics plus NSAIDs was associated with an increased rate of AKI (ratio 1.31, 95% CI 1.12–1.53), the highest risk observed in the first 30 days (ratio 1.82, 95% CI 1.35–2.46) [102]. ACE inhibitors or ARBs can induce a decrease in glomerular filtration via vasodilation of the efferent renal arteriole. Diuretics may contribute to AKI via hypovolemic hypoperfusion. And all NSAIDs, including COX-2 inhibitors, have been associated with an increased risk of AKI, due to the blockade of the COX-1 and COX-2 enzymes preventing prostacyclin synthesis, causing afferent arteriolar vasoconstriction [102,103]. There is pronounced risk, e.g., in patients with hypovolemic states, and after 3–7 days at maximal inhibition from NSAID steady-state plasma concentrations [103]. IPM aimed to avoid the triplet entirely. Besides the discontinuation of NSAIDs, except for individual indications for prevention of heterotopic ossification, IPM also focused on necessary dose reductions of identified ACE inhibitors adjusted to the patient’s individual eGFR. In addition, adequate and cardiac-balanced fluid supplementation from the beginning of hospitalization was always a major target of trauma care professionals. 

IPM addressed the high prevalence of anemia in our elderly patients and differentiated iatrogenic drug-induced anemias, either hematopoietic or hemorrhagic side effects, also cumulative by different drugs or enhanced by pharmacodynamic or pharmacokinetic DDIs, for reasonable targeted treatment.

Apart from renal diseases, major risk factors and most frequent triggers for CKD onset and progression are arterial hypertension and diabetes mellitus [104]. However, for the acute renal decline, as measured by redGFR, we did not find an association with either of these factors during hospitalization. This is because we had a very short observation period for each patient only and addressed adequate blood pressure and diabetes management in the hospital setting.

For patients with severe acute medical conditions such as surgical or cerebrocardiovascular with metabolic diseases, optimizing prescribing would make much more sense than tightening up the demand for deprescribing because elderly patients often suffer from multiple serious comorbidities, each of which requires adequate drug therapy. As a result of IPM, it is most important and highly effective to address the entire ADRs, cumulative ADRs from pharmacodynamic DDIs, to exclude any undesired increase in drug bioavailability and risk of toxicity by pharmacokinetic DDIs, and to accurately adjust the dose according to the hepatic and renal organ function for drug metabolism and excretion.

We documented the effect of IPM via different statistical models. The significant association with the decrease in redGFR was even maintained in the multivariable regression analysis for the overall IPM frame including patients before the 14 daily IPM-visit without an individual medication analysis. Yet, it is important to address, that these patients outside the IPM context remained particularly at risk of missed dose adjustment to renal function with the onset of antibiotics. Whether these cases result from lack of knowledge, disregard of renal function, or operational work stress remains questioned but indicates necessary efforts to consequently enhance attention in this particular respect. Independent of too high a dosage in antibiotics as a risk of nephrotoxicity in our study, an extensive spectrum of medications poses nephrotoxic ADRs. ADR from drug-induced nephrotoxicity remains one of the most common causes of acute kidney injury among hospitalized patients, such as from antimicrobials like antibiotics known to induce structural and functional renal impairment [79].

The KDIGO AKI Guideline recommends that all people with CKD be considered at increased risk of AKI, which is further increased with intercurrent illness, investigations, and procedures. For most elderly patients admitted to a trauma center with fractures requiring investigation and surgical treatment, this is precisely the constellation that must be considered.

Accordingly, the IPM adhered to the KDIGO recommendations in the context of riskful contrast media [105], and indicated bicarbonate supplementation in metabolic acidosis in the absence of contraindications [105,106,107], e.g., reduced respiratory CO2-eliminating capacities. Perioperative renal protection from AKI in patients taking an increased number of drugs during hospitalization in addition to their preexisting outpatient medications appears to be a matter of adequate and preferably individualized prescribing. The complete prevention of redGFR in the timely framework of IPM even in advanced CKD underlines that the risks of drug therapy are the most important component of CKD progression or AKI during perioperative hospitalization, e.g., in post-major orthopedic surgery [108], with drug-induced nephrotoxicity being more frequent among e.g., elderly patients and in specific clinical conditions [109], apparently entirely preventable by our IPM measure. 50.8% CKD under-reporting with alarmingly high rates in geriatric compared to nephrology departments (71.1% vs. 10.2%, *p* < 0.001) and simultaneous prescription of inadequate nephrotoxic agents at discharge demonstrates the cross-sector expansion of this high-risk problem, particularly for the elderly and, on this aspect profoundly susceptible patients [110]. The International Group for Reducing Inappropriate Medication Use & Polypharmacy (IGRIMUP) recommends a shift away from the current focus on single diseases to one that simultaneously addresses multiple diseases and patient priorities [111]. As from our study results, this worldwide challenge obviously can be completely resolved with effective IPM, designed for the same goal and the individual patient-centered and inherent socioeconomic purposes.

CKD is a well-recognized risk factor for AKI, which itself in turn accelerates CKD itself, and accordingly, AKI requires optimized care [112]. The incidence of AKI varies from 5.0% to 7.5% in hospitalized patients and reaches up to 50–60% in critically ill patients. AKI is known to complicate the perioperative course for even up to 50% of surgical patients, especially. It is still rated as one of the most frequently underdiagnosed and undertreated postoperative health complications, and it should be addressed by clinical prediction scores and biomarkers [113], renal functional reserve assessment and a personalized multidisciplinary approach [114], of which the latter is provided by our IPM. A systematic literature review and meta-analysis on additional pharmacological drug treatment interventions for the prevention of renal injury in surgical patients showed some clinical benefit for improved renoprotection e.g., atrial natriuretic peptides analogs, inodilators, and vasopressors in particularly vulnerable ICU patients predominantly [115]. 

Because the incidence of AKI has increased in the last decades, and patient age is an important non-modifiable risk factor due to the physiologic decline of GFR and impaired renal reserve [116], we need to pay special attention to this increasing risk group for prevention strategies. Furthermore, AKI and CKD have become addressed as interconnected syndromes [117]. This supports our recommendation to implement IPM in all patients at risk as a preventative and effective tool for both, redGFR avoidance and prevention of CKD onset or progression. Among the often more patient-related risk factors associated with postoperative mortality, such as age, hypertension, diabetes mellitus, cardiac failure, and preexisting CKD, compared with surgery-associated factors such as decreased renal perfusion or, e.g., enhanced intra-abdominal pressure during major abdominal surgery, preexisting CKD contributes to the highest rate of AKI requiring dialysis in cardiac surgery patients [118]. Coding for acute renal failure (ARF) in the United States was increased with age, male sex, black persons, CKD, congestive heart failure, chronic lung disease, sepsis, and cardiac surgery. It was associated with a 2-day increase in hospital length of stay (LOS) (*p* < 0.001), 4-fold hospital mortality and 2-fold discharge to post-hospitalization care, accounting for serious patient harm and increased health economic resource utilization [119]. 

In the inpatient setting, inappropriate dosing accounts for up to 42% of medication errors, ranking it as one of the most important preventable risk factors. In addition to inappropriate medication dosing, advanced age, CKD, and the number of medications were evaluated as major risks for prescription errors. The substantial degree of incidental adverse events and negligence in hospitalized patients increased highly significantly with age. Rates of adverse events differed significantly across specialties, while negligence remained the same [120]. The Harvard Medical Practice Study II analyzed the nature of adverse events in hospitalized patients, finding that drug complications, accounting for 19% were the most common cause of adverse events compared to others. According to the authors 32 years back, the reduction in adverse events “must wait for the improvement of medical knowledge”, the identification of their causes and the development of prevention methods [93]. The question is whether we have succeeded in achieving this over the last 3 decades. The problem may even have become intensified with the global aging of patients on polypharmacy without the development and implementation of reliable and potent preventative strategies. Despite its limited power as a retrospective study, IPM seems to be the reliable preventative measure in this long overdue and very challenging context.

Several cardiovascular agents, besides antibiotics and NSADIs, have been identified as the most common causes of drug-induced kidney injury [121].

In this context, the IPM also referred to statin doses in the individual elderly with regard to the entire medication list and patient condition. Patients with high potency statin treatment defined as ≥10 mg rosuvastatin, ≥20 mg atorvastatin, and ≥40 mg simvastatin, also often administered in the outpatient medication of our patient population, were hospitalized for AKI within the first 120 days from treatment start. This was 34% more than in the reduced doses in low potency statin treatment. The rate was even enhanced in patients without CKD [122], probably indicating statins as a possible trigger for the onset of CKD. A further study confirmed this enhanced AKI risk for higher potency simvastatin [123]. 

As a drug, SGLT2 inhibitors, which have emerged as disease-modifying agents with benefits not only in CKD, number needed to treat (NNT) = 19, always pose ADR and DDI risks [124,125]. In comparison, the similarly high efficacy of IPM with an NNT = 18 is another, but very different, effective preventive measure to avoid the progression of CKD without exposing the patient to additional medications, contraindications, ADRs, and DDIs. Knowledge of age-related physiologic changes resulting from alterations in various biological domains and the associated effects on the pharmacodynamic and pharmacokinetic actions of drugs should sensitize the physician to fine-tune each medication prescribed [126]. Increasing the necessary awareness of preexisting CKD may be achieved by supplying a current eGFR with each medication list of the patient. This should become mandatory.

The obviously high impact from IPM indicates a strong effect of drugs on the progression of renal impairment in the elderly population. Whether this applies only to hospitalized patients or also to elderly outpatients remains to be investigated accordingly but may be assumed. The fact, that the IPM results are impressive despite the inevitable increase in polypharmacy with age shows that the positive effect on redGFR can be achieved by comprehensive individual medication management alone, rather than by a necessary reduction in the number of medications.

## 5. Strengths and Weaknesses

This is a retrospective controlled clinical study with all its inherent limitations. It involves two sets of data collection conducted by the two same investigators for both. The patient records and datasets used were not explicitly designed for the study, and data on variables may be missing. Focusing on a wide range of possible associated variables risks more gaps in the findings. This is equally true for both, the CG and the IG. Randomly achieved highly matched groups between CG and IG minimized potentially relevant confounders and supported comparability. Although redGFR had already occurred at the time of recruitment, the study was not biased by knowledge of outcome status because both cohorts included samples blinded to the outcome. Since the progress of renal impairment was not reliably documented and coded in the patient discharge letter, we referred to a robust and most concise manifestation of further renal impairment with a further reduction of eGFR ≥ 20 mL/min throughout the in-hospital course as a strong criterion. The reference to eGFR values on admission to estimate CKD stages lacks long-term follow-up values and measurements of albuminuria. Both additional aspects for accurate calculation of possible progressive CKD stages were not applicable in the study in geriatric traumatology due to the unavailability of contemporaneous amounts of proteinuria/albuminuria and short perioperative hospital stay with a primarily surgical focus on fracture treatment. Another limitation was the laboratory calculation of eGFR values using the MDRD formula, which becomes less accurate, especially with increasing age, in the majority of our study population, without further subdivision of eGFR values above 60 mL/min/1.73 m^2^. The concept of this study was the trigger for the introduction of eGFR CKD EPI in UKH for further assessments. We did not consider the prevalence or severity of certain conditions, such as the extent of possible blood pressure or glucose fluctuations or the severity of diabetes stage, tobacco or alcohol use, diet, or physical activity. Nevertheless, these remaining confounders are unlikely to explain the uniform strong IPM effect observed in reduced redGFR.

There was only one medication reviewer, with education and experience in internal medicine, nephrology, and clinical pharmacology. Her educational background may be an advantage in analyzing the drug list in a conceptual manner in more detail than clinical pharmacists or pharmacologists can do. Despite these being medication analyses of a single individual, the determinants of medication review are reproducible and clearly defined by all patient and medication scores depicted. The IPM internist has applied their decades of kidney transplant experience in treating similarly vulnerable kidneys of geriatric and intensive care patients analogously to the concept of this IPM.

Although the data refer to a selected patient operative ward under hospital conditions, they may reflect the typically elderly patient conditions we need to treat nowadays, as they cover all out-hospital medications and reflect the real-world risk from uncontrolled polypharmacy complexities that lead to red eGFR and other organ injuries, fall events necessitating trauma surgery and risk for delirium and cognitive decline. IPM as an individual medication review that is conducted as intensively as possible from a synoptic internistic and clinical pharmacologic view does not consider genetic pharmacological aspects additionally. We did not measure blood values that attribute ADRs to iatrogenic medical agents due to elevated levels. We also did not include and adjust for intraoperative parameters such as type of operation for trauma, operation duration, severe prolonged blood pressure drops, and blood loss, which would have been of further interest. The head of geriatric traumatology assures that there have been no relevant changes in the intraoperative procedure for the treatment of all injury entities during the entire study period. Thus, from a surgical perspective, there are no structural reasons to expect a change in blood loss. The individual course was not assessed additionally.

The more than 8 years of daily IPM experience with a critically ill or multimorbid elderly interdisciplinary patient population covers a particularly vulnerable group with polypharmacy and, partly, already reduced organ function. There is almost no possibility to clearly identify the entire frequency of ADR manifestation, but the more vulnerable the patient is as a result of preexisting illnesses, organ deterioration, and polypharmacy, the higher the ADR risk. The spectrum of ADRs and DDIs addressed is based primarily on the SmPCs. Their completeness and, in some cases, even the clearly defined metabolic pathways of active substances and risks from inactive metabolites are not always fulfilled, thus leaving out still unknown grey areas, which themselves may be additionally dangerous for the treated patients. 

It is a practice-based study with many years of continuity and experience. Because throughout the observation period, both, the senior traumatologist and the IPM physician remained the same, the recordings in the database can be considered fairly accurate and consistent. This is the first clinical study to document a strong association of medication review-based medical adjustments with the complete prevention of redGFR. The study topic addresses an urgent and demographically increasing public health problem, and IPM emerges as a compelling prevention tool that is still underrepresented. With the IPM, a continuity of interdisciplinary cooperation has been established in which the patient-oriented optimization of acute treatment in geriatric traumatology is always at the forefront. The continued focus on avoiding drug-induced risks and individually considering DDIs and overdosages is likely to have had an additional superimposed positive systemic class effect over the years. The compelling pilot data from our retrospective study on IPM efficacy support the feasibility of designing a future, more powerful study including a quantitatively larger experimental group to obtain more explicit statements and conclusions about IPM effect on redGFR. Ideally, a prospective, randomized controlled trial involving multiple medical disciplines would be beneficial to substantiate our findings and examine the extensively established IPM procedure in multiple settings to make further reliable conclusions about additional predisposing factors. But this raises ethical concerns in an effort to provide optimal care for all elderly patients entrusted to us.

## 6. Conclusions, Challenges, and Outlook

We have to accept that polypharmacy is almost unavoidable in elderly multimorbid adults, who often dispose various cerebrocardiovascular and organ diseases in the presence of metabolic and endocrinologic disorders. At the same time, especially these are the patients already suffering from age-related organ impairment that affects drug elimination capacities on the drug metabolism and excretion level. Given this background, despite high-level polypharmacy, the applied IPM consequently adapted to the patient’s very individual clinical condition, was associated with the potent prevention of iatrogenic redGFR through the appropriate prescription of each drug, taking into account all clinically relevant dose adjustments, ADRs, DDIs, missing prescriptions and contraindications from the individual drug list. With an optimized and overdue real-world drug and patient safety through pharmacovigilance, the same IPM does not only seem to prevent renal impairment and progression of CKD. It is also associated with the impressive reduction in complicating delirium [8] and fall events (in preparation to publish) and benefits the course of the ICU patients [17]. 

The documented severe discrepancy in manifest CKD versus diagnosed CKD contributes to a serious additional risk of overdosage in this high percentage of patients treated, re-transferring the unawareness to the ambulatory setting again. Awareness of renal impairment is the sine qua non for examining the appropriateness of a drug’s dose. Therefore, the doses are much more often too high than realized. The SmPC suggested normal dose may already be too high in elderly patients is unfortunately referenced in only a few drug SmPCs. Although the vast majority of drug consumers are elderly patients, drugs remain generally approved in healthier younger adults with regular organ function and without the broad spectrum of concomitant diseases, contraindications, and comedications that can exacerbate ADRs in the elderly per se through increased susceptibility at the level of pharmacodynamic action and, cumulatively, and through DDIs. 

To compensate for and overcome these deficiencies and challenges, IPM seems an important preventive tool with its high impact on avoiding further renal impairment by drugs, even in very old patients over 80 years of age undergoing surgical intervention. Because redGFR associated with an eGFR ≥ 60 mL/min/1.73 m^2^ as well as with an eGFR < 60 mL/min/1.73 m^2^ was completely eliminated by IPM, the IPM effect may be preventive for both, iatrogenically induced onset of renal impairment and progression of manifest CKD. Therefore, it should be performed as early as possible and at regular intervals in all geriatric patients on a mandatory basis to prevent drug-induced and drug-enhanced renal injury. As a further patient and socioeconomic benefit, IPM also reduced the necessity of intensive care and hemodialysis. 

The available electronic patient record supports IPM in optimizing safe and highly individualized prescribing, a task that digital tools currently are unlikely to cover as comprehensively. Yet, recording a CKD diagnosis in the medical record may at least help uncover potentially inadequate prescribing through clinical decision support. 

Given the pressing situation of CKD management, we should not only provide recommendations for specific drugs but advocate IPM for the entire drug list. For future direction, the extensive evidence resulting from more than 58,600 IPM self-conducted analyses from a daily broad-based polypharmacy real-world dataset corresponding to the current commonly prescribed drug combinations in older adults will be digitized to provide the specified drug risks directly with the use of the electronic patient record.

A safe healthcare system inevitably demands eliminating any type of drug-related patient harm. To urgently prevent the drug-induced iatrogenic onset or progression of CKD in the increasing elderly patient population with polypharmacy worldwide, the study insights and effects require recommendations at both, the individual patient and the policy levels: It should become mandatory to document updated eGFR with each medication list and to ICD-code drug-induced renal injury as a separate diagnosis to increase physician awareness of this iatrogenic condition and to perform mandatory comprehensive IPM on a regular basis. Available guidelines are deficient in adequately addressing and compensating for the risk of polypharmacy for the onset or progression of CKD.

## Figures and Tables

**Table 1:** Variables collected for data analysis aimed at addressing various clinical research questions in 404 ≥70-year-old hospitalized trauma patients.

**Demographics:** age, gender, type of residence (home/nursing home)
**Vital parameters at admission:** BMI, blood pressure (day course), heart rate (day course)
**Continuous and acute medication:** number of drugs, angiotensin converting enzyme inhibitors (ACE inhibitors), angiotensin receptor blockers (ADRs = sartans), calcium antagonists, differentiated ß-blockers, α-blockers, antibiotics, antifungals, antiarrhythmics, antidementives, anticonvulsants, different oral anticoagulants, bisphosphonates, different antiplatelet drugs, different diuretics, antipsychotics, antidepressants, St. John’s wort, oral antidiabetics, insulin, antiparkinsonian drugs, benzodiazepines, proton pump inhibitors (PPI) (incl. dosage), ophthalmics, urological drugs, muscle relaxants, opioids, “Würzburger pain drip“ ^1^, tramadol, non-steroidal anti-inflammatory drugs (NSAIDs), further analgesic agents, antiemetics, thyroid hormones, xanthines, uricosurics, uricostats, statins, vitamin D, corticosteroids, other drugs (e.g., hormones, cytostatics)
**Laboratory parameters at admission:** blood count, electrolytes, inflammation parameters, estimated glomerular filtration rate (eGFR) during course of hospital stay, GFR BIS-formula, serum creatinine, myoglobin, coagulation parameters, urinalysis, bacteriuria
**ECG (if available online):** rhythm, frequency, QT interval ^2^, atrioventricular block (AV block)
**Diagnoses of interest ^3^:** arterial hypertension, heart failure, complicating delirium, cognitive impairment to dementia, Parkinson’s disease, further central nervous system (CNS) disorders, chronic obstructive pulmonary disease (COPD), diabetes mellitus, osteoporosis, chronic kidney disease (CKD)
**Additional course aspects:** changes in laboratory findings, blood pressure, heart rate, temperature, cognitive changes/disturbances, pain symptoms and profile, other subjective complaints of the patient
**Other parameters:** acute admission injury, operation, transient stay in IMC ^4^ or ICU ^5^, acute and chronic hemodialysis, length of hospital stay, perioperative infections requiring antibiotics, fall risk scale according to Huhn (0–31 points, broken down according to: age, mental status, excretion, history of falls, gait/balance, activities, medication, alcohol), pacemaker, defibrillator, contrast medium application.

^1^ Combination of tramadol, metamizole, and metoclopramide administered intravenously or partially orally ^2^ time from the start of the Q to the end of the T wave (ECG) ^3^ coded in the hospital discharge letter ^4^ Intermediate care ^5^ Intensive care unit.

**Table 2:** Prevalence of renal impairment with eGFR <60 mL/min/1.73 m^2^ (MDRD) at hospital admission, comparing control (CG) and intervention group (IG) (absolute numbers and %).

Renal Impairment at Admission *	CG *n* = 199	IG *n* = 204
**eGFR <60 mL/min/1.73 m^2^**	**83 (41.7%)**	**101 (49.5%)**
CKD 3a	38 (19.1%)	47 (23.0%)
CKD 3b	24 (12.1%)	33 (16.2%)
CKD 4	17 (8.5%)	13 (6.4%)
CKD 5	4 (2.0%)	8 (3.9%)

* Since prior to this study, until 3/2016, eGFR ≥ 60 mL/min/1.73 m^2^ was only estimated as one category by the MDRD, we could not further differentiate between CKD 1 and CKD 2 for patients with eGFR ≥ 60 mL/min/1.73 m^2^.

**Table 3:** Comparison of mean eGFR MDRD versus mean BIS-1 values, standard error and standard deviation in the severe and moderate renal dysfunction.

	Patient Number	Mean	Standard Error	Standard Deviation
BIS-1-formula	42	21.89	1.08	7.01
eGFR (0–29.99 mL/min)	42	20.01	1.10	7.16
BIS-1-formula	142	42.21	0.70	8.38
* eGFR (30–59.99 mL/min)	142	46.75	0.71	8.49

* Since prior to this study, until 3/2016, eGFR ≥ 60 mL/min/1.73 m^2^ was only estimated as one category through the MDRD formula, we could not further compare eGFR and BIS-1 for patients with eGFR ≥ 60 mL/min/1.73 m^2^.

## Data Availability

The datasets generated and analyzed for the current study are available from the corresponding author on reasonable request.
